# Effectiveness of Osteopathic Treatment on the Spinal Column as Measured by the Spinal Mouse®: A Case Series

**DOI:** 10.7759/cureus.28074

**Published:** 2022-08-16

**Authors:** Rocco Colati, Alessandro Pagano

**Affiliations:** 1 Osteopathy, Independent Researcher, Ascoli Piceno, ITA

**Keywords:** spinal column height, vertebral mobility, spinal column, osteopathic manipulative treatment (omt), spinal mouse

## Abstract

Introduction

This case series aims to compare changes in spinal column mobility using the Spinal Mouse® (Idiag, Volketswil, Switzerland), a device providing a spatial survey of a single vertebra's position. The measurements have been made before and after an osteopathic treatment with different spinal column positions on healthy subjects. We presumed that osteopathic treatment is able to improve spinal column mobility.

Methods

The measurement was carried out with the naked spinal column in the following positions: standing, static bending, dynamic bending, and conducting the static Matthiass test, which consists of maintaining the standing position with outstretched arms, with a 1 kg weight. We evaluated the vertebral tilt degrees and changes in height (expressed in mm) in the mentioned positions before and after the osteopathic treatment.

Results

We observed improvements in spinal column tilt (expressed in degrees) and in vertebral metameres length (expressed in mm) in a standing position (T<0.04 and T<0.04). We also noted a global increase in tilt (expressed in degrees) during the static bending position (T<0.05) and in the thoracic tract during the Matthiass test (T<0.02).

Conclusions

The present study highlighted that osteopathic treatment was able to increase vertebral mobility, concerning tilt (expressed in degrees) and length (expressed in mm), evaluated in different positions.

## Introduction

According to the World Osteopathic Health Organization (WOHO), osteopathy is an established and recognized system of preventive healthcare which uses a manual approach for diagnosis and treatment. It is based on a set of evaluations in which the patient is examined from a global point of view [1]. It’s impossible to relegate osteopathy to a single field of application. For that reason, it is described as a complex intervention based on multifactorial diagnostic work [2-3].

The osteopathic treatment role in the modification of morphological and functional parameters of the spinal column has been examined all over the years. Brandl et al. have demonstrated in their study that the osteopathic treatment leads to a modification in the kyphotic angle, but not in the lordotic angle, without specifying the particular spinal column tract on which the study was conducted [4]. However, there are no studies in the scientific literature that comprehend the sacral tract and mention any device used for an objective assessment of the angle of the examined curves.

The spinal column has numerous afferent fibres, from an anatomical and neurophysiologic point of view [5-6], so it is necessary to be more specific about diagnostics and therapies. In the area of the spinal cord, in fact, in response to even minimal afferent stimulus (for example traumas or peripheral somatovisceral irritation), a neurologic hyperactivation associated with sympathetic hypertonia can be seen. This event has been studied over the years by several authors such as Irwin Korr and Cervero, who stated that trauma, even if not particularly substantial, could cause modifications in the neurologic reaction of the dorsal horns of the spinal cord, as a result of an increase in input influx from neuromuscular spindles, followed by a decrease in neuronal excitability limit. The basal activity of the spinal cord increases as a consequence of a hypersensitivity and excitement condition, transforming the spinal cord into a “facilitated segment”, i.e. an area in which we can see a change in muscular paraspinal tissues, related to the organs innervation metameres, resulting from a physiological alteration that can be recognized to the palpation [7-8]. This phenomenon can lead to an alteration of articular mobility and physiological range of motion. For these reasons, it’s important to highlight the fact that the spinal column has within itself countless afferent fibres that can also influence spinal column mobility.

It has been shown that osteopathic treatment can normalize this hyperactivation in the autonomous nervous system [9]. Osteopathic treatment aims to normalize the correct expression of this system, whose task is to regulate therapeutical physiological processes, ensuring a good global functionality of the organism.

The Spinal Mouse® (Idiag, Volketswil, Switzerland) is a non-invasive, computerized method of analysis, validated in scientific literature, able to assess the curves and the mobility of the spinal column [10-11].

Thus this study tries to evaluate the effect of osteopathic treatment on the spinal column in six healthy subjects before and after the osteopathic treatment, with specific attention to the sacrum. It is possible using the Spinal Mouse which allowed us to detect the segmental mobility parameters of the spine.

## Materials and methods

We examined six healthy subjects (three females and three males), aged between 29 and 44. The exam has been made before and after an osteopathic treatment, without considering the patient’s initial clinical condition, which constitutes a test-retest study.

The survey was made using the Spinal Mouse, which allows one to recreate a static and dynamic mapping of the spinal column through the software Idiag without any kind of radiation. This device can accurately recreate the spinal column, using the vertebral spinous processes as anatomical landmarks. The collected data are sent via Bluetooth to the software that saves and analyzes the collected parameters.

The Spinal Mouse is manually moved by an operator along the patient’s spinal column, from the spinous process on C7 to the median sacral crest, at the S3 level [12].

The exam is made in different positions: standing upright in a neutral position; standing upright with the maximum stretch; standing in a position of maximum forward bending [13].

The measurement was made on the naked back, in a standing position, then in maximum forward bending (with no bend knees) and, a second time, using the Matthiass Test, with a weight of 1 kg. This test shows information about neuromuscular activation of core muscles. It must be done while maintaining an upright position, with outstretched arms and with a neutral rotation of the upper limbs. Every arm carries a 1 kg weight for 30 seconds. Next, the operator makes a new scan with the Spinal Mouse, while the patient still carries the same weight. This scan controls the muscles that stabilize the spinal column [14].

At the end of this operation, the software saves the segmental mobility parameters of the spinal column. The device showed consistent high reliability [12].

In the next phase, the osteopathic exam was made always by a single operator, in order to highlight the somatic dysfunctions, i.e. alterations in tissues quality (resulting from a district metabolic alteration), in addition to alterations in placement and movement of a body segment, connected or not to local pain.

The treatment has taken place using indirect techniques called balanced ligamentous tension release (BLT) [15]. This technique has two steps which are disengagement (at the joint in question) and balance. This last one involves feeling for release in the form of a change in the palpatory quality of the structure being treated indicating that the ligaments involved in the dysfunction have been rebalanced. The change can be i.e. a change in skin tension, temperature, or muscle tension. At the end of the diagnostic-therapeutical phase, the operator has made a new scan with the Spinal Mouse.

The osteopathic exam on the patients was carried out following the Johnston Functional Method, which is based on the body response at the operator’s solicitation. In order to complete the exam, intrinsic and extrinsic strength tests have been used. These evaluation methods have not yet been validated (there is no research in this direction), but they represent an osteopathic evaluative approach derived from the scientific literature on PubMed, which can be considered as a suggestion and starting point for comparing initial and final results by the clinician.

## Results

The aim of the present study was to detect postural modifications brought about by an osteopathic treatment, using the Spinal Mouse as a tool already validated in other scientific works. All of the six healthy subjects received both evaluations, before and after the treatment.

The parameters given by the two measurements are shown in Table 1, where data were grouped together, with the patient maintaining the following positions: in the standing position the measurement analyzes the orthostatic position of the patient, in the static forward bending position the measurement assesses the maximum forward bending of the patient; with the Matthiass test the measurement assesses the neuromuscular stabilization of the axial skeleton in static conditions, in the dynamic bending position the measurement is made while the patient moves from a standing position to a forward bending position, with the dynamic Matthiass test measurement recreates the neuromuscular stabilization of the axial skeleton in dynamic conditions. The analysis was performed with an Excel spreadsheet using statistical formulas to calculate the median, the confidence interval, and the Student's test (t-test), the results of which are represented in the tables (Tables 1-5).

**Table 1:** Standing position analysis, before and after the treatment. Negative values show lordosis angles. Positive values show kyphosis angles. Q1: 25% confidence interval Q2: 75% confidence interval

Standing position		Without treatment						With treatment				T-test
		Q1		median		Q3		Q1		median	Q3	
Tilt		2.25		3.5		4.75		3.25		4.5	6.5	0.04
Lumbar		-8.25		-4		-0.75		-8.25		-4	-1.75	0.42
Sacrum/hip		12.25		14		15		11.5		13.5	20.75	0.55
Thorax		2.25		4		5.75		3		5	6	0.09
Length		486.5		510		527.5		507.75		521	552.25	0.04

**Table 2:** Static forward bending analysis, before and after the treatment. Negative values show lordosis angles. Positive values show kyphosis angles. Q1: 25% confidence interval Q2: 75% confidence interval

Static forward bending	Without treatment			With treatment			T-test
	Q1	median	Q3	Q1	median	Q3	
Tilt	86.5	105	106.75	94	108.5	115.5	0.05
Lumbar	3	5	7	4	5	7	0.42
Sacrum/Hip	62	67	69.5	57	66	75.75	0.98
Thorax	4.25	6	7.75	4	6	8	1
Length	562	619	636.25	562	619	536.25	0.42

**Table 3:** Static Matthiass test analysis before and after the treatment Negative values show lordosis angles. Positive values show kyphosis angles. Q1: 25% confidence interval Q2: 75% confidence interval

Static Matthias test	Orthorstatic without treatment			Orthorstatic with treatment			T-test
	Q1	median	Q3	Q1	median	Q3	
Tilt	1	-0.5	2.25	-3.75	-2	1.25	0.18
Lumbar	-7	-4	0	-8	-5	1.75	0.93
Sacrum/hip	9	15.5	19	12	13.5	16.5	0.80
Thorax	2.25	5	6.75	1	4	6	0.02
Length	507	530	555.25	484	512	563.25	0.37

**Table 4:** Dynamic forward bending analysis, before and after the treatment Negative values show lordosis angles. Positive values show kyphosis angles. Q1: 25% confidence interval Q2: 75% confidence interval

Dynamic forward bending position		Without treatment						With treatment				T-test
		Q1		median		Q3		Q1		median	Q3	
Tilt		101		101		103.25		90.5		102.5	112.25	1
Lumbar		4		10		12.5		5.75		9.5	13.25	0.41
Sacrum/Hip		45.25		48		60.5		41.5		47.5	58.75	0.17
Thorax		-1.75		2		4		-1		1	5	0.73
Length		66.5		106.5		115.75		29		60	108.25	0.12

**Table 5:** Dynamic Matthiass Test Analysis, before and after the treatment Negative values show lordosis angles. Positive values show kyphosis angles Q1: 25% confidence interval Q2: 75% confidence interval

Standing dynamic Matthiass test	Without treatment			With treatment			T-test
	Q1	median	Q3	Q1	median	Q3	
Tilt	-4.75	-3	-0.5	-8.25	-2.75	112.25	0.44
Lumbar	-3	-1	1	-2	1.25	12.25	0.27
Sacrum/hip	-3.75	2	7	-7.5	1.75	58.75	0.47
Thorax	-1	0	2	-1.75	1	5	0.42
Length	10	22	32.5	-28.25	10.75	112.25	0.80

The analyzed data showed that osteopathic treatment brought about relevant changes in every posture. In four cases, the p-value was <0.05. This value refers to the measurement in the standing position, particularly regarding “tilt” parameters (expressed in degrees), “length” parameters (expressed in mm), in the Matthiass Test (mainly in the thoracic tract of the spinal column), and in the parameter referring to the tilt in the static forward bending position.

**Figure 1:**
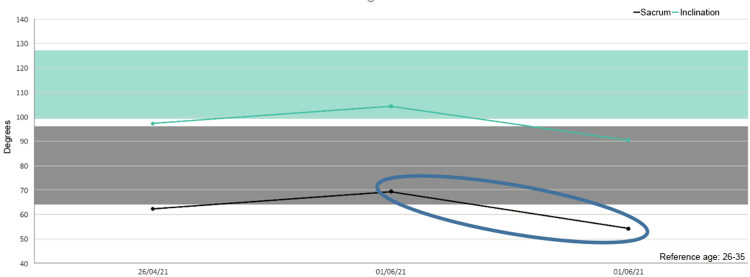
Sacrum mobility before and after the treatment on the examined patients

**Figure 2:**
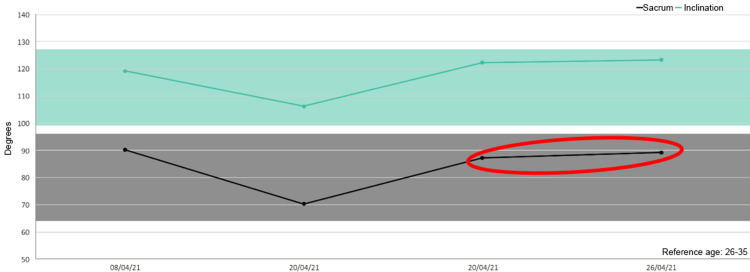
Sacrum mobility before and after the treatment on the examined patients

**Figure 3:**
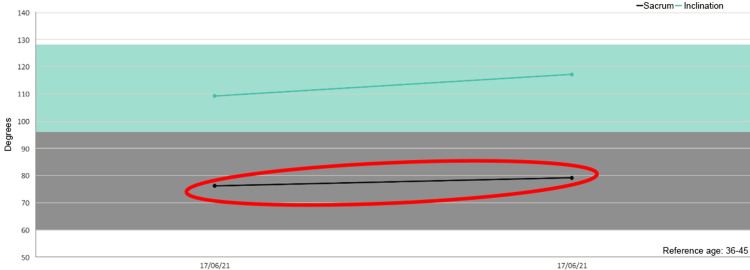
Sacrum mobility before and after the treatment on the examined patients

**Figure 4:**
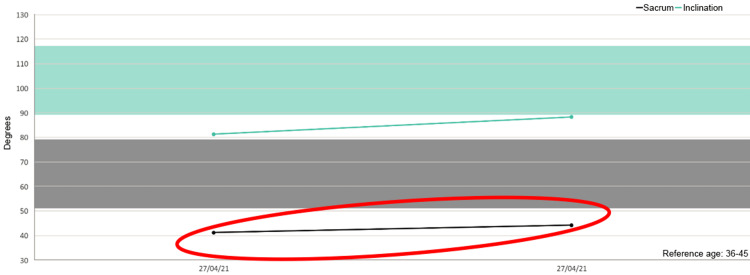
Sacrum mobility before and after the treatment on the examined patients

**Figure 5:**
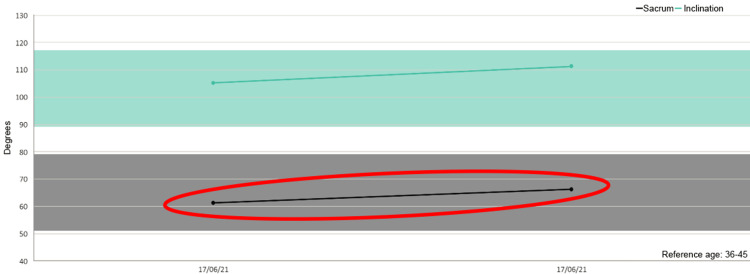
Sacrum mobility before and after the treatment on the examined patients

**Figure 6:**
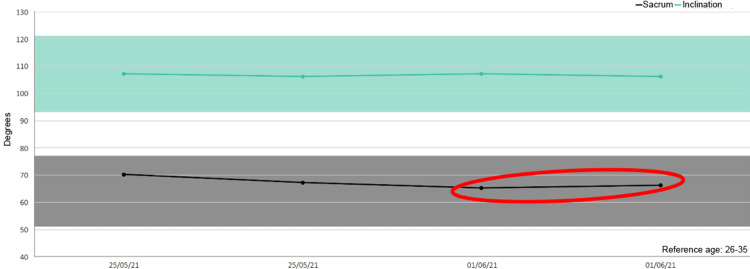
Sacrum mobility before and after the treatment on the examined patients

Besides, in five out of six patients we detected increased mobility of the sacrum after the treatment as shown in the figures (Figures 1-6).

## Discussion

The Spinal Mouse allowed us to examine in particular the morphological sphere of the spinal column. The treatment effectiveness was made quantifiable by the changes detected thanks to the device. Assuming that in the sacrum area there are a large number of parasympathetic ganglions, that the parasympathetic sacral system provides innervation to the visceral part in the lower abdominal cavity (starting from the transverse colon’s third distal) and in the pelvic area, that preganglionic axons sprout from spinal cord sacrum segments (run from the 2nd, 3rd, 4th and 5th sacral nerve) forming sacral plexus and pudendum plexus and that there are very strict connections among bone, neurological and visceral components, we can deduce that altered mobility in sacral tract affects the physiological action of the autonomic nervous system. This is mainly relevant to etiopathogenesis and in maintaining somatic dysfunction [16-17].

With the help of the Spinal Mouse, we were able to note that four measured parameters showed an improvement. The first two parameters are referring to tilt, expressed in degrees and length (expressed in mm) of the spinal column, in standing position, and the third parameter is referring to the spinal column tilt in static forward bending. Research in scientific literature talks about progressive reduction in spinal column height detected thanks to two exams during the same day [18], but the present study’s results show that osteopathic treatment is able to reverse the trend. The data collected in the present research, however, concur with scientific literature [19] about spinal column flexibility. Indeed, the same article shows that spinal column flexibility increases from morning to night. This result can be overlapped with our study's results obtained after the osteopathic treatment. The only variance lies in the distance in time between the two exams. In the mentioned studies, the two measurements were made respectively in the morning and in the night of the same day, several hours later. In the present study, however, we made the second exam an hour after the first; during this hour an evaluation and an osteopathic treatment have taken place.

The fourth, very promising parameter is the Matthiass Test. Even if the clearest improvement was detected in the thoracic tract, this parameter leads to thinking that osteopathic treatment is able to induce an adequate functional recovery in the spinal column.

Several studies in the literature show how osteopathic treatment and manual therapy can reduce the neurogenic inflammation degree [20-21]. The research states that treatment has effects on the physiological activity of fibroblasts, which moderate pro or anti-inflammatory cytokines. There are also studies in the literature that highlight the bond between fibroblastic activity, pain, and articular mobility [22]. As a consequence, even if we couldn’t find scientific evidence proving in a direct manner the interaction between spinal column mobility and neurogenic inflammation, we could assume that the link between these two aspects is due to fibroblastic activity, which is normalized thanks to osteopathic treatment.

This study has some limitations. First of all, the number of subjects analyzed is low and this aspect can generate misunderstandings in the interpretation of the results. Furthermore, the tool does not allow the assessment of the cervical tract, which would provide better and global screening of the subjects examined.

## Conclusions

With osteopathic treatment, as much as it could be taken as a passive solicitation, the entire patient’s organism activates healing processes with a minimum stimulus by the operator. We can therefore assume that the metabolic-metameric rebalance can be associated with an optimal reaction from a functional point of view. It is certainly necessary to expand the sample to give greater statistical significance to the report and to better understand the underlying process.
